# The mixed benefit of low lipoprotein*(a)* in type 2 diabetes

**DOI:** 10.1186/s12944-017-0564-9

**Published:** 2017-09-12

**Authors:** Michel P. Hermans, Sylvie A. Ahn, Michel F. Rousseau

**Affiliations:** 1 Division of Endocrinology and Nutrition, Cliniques universitaires St-Luc and Institut de Recherche Expérimentale et Clinique (IREC), Université catholique de Louvain, Avenue Hippocrate UCL 54.74, B-1200 Brussels, Belgium; 2 Division of Cardiology, Cliniques universitaires St-Luc and Pôle de Recherche Cardiovasculaire, Institut de Recherche Expérimentale et Clinique (IREC), Université catholique de Louvain, Brussels, Belgium

**Keywords:** Lipoprotein*(a)*, Diabetes, Insulin secretion, Microvascular, Atherogenic dyslipidemia, CAD

## Abstract

**Background:**

Lipoprotein*(a)* (Lp*(a)*), a variant low-density lipoprotein (LDL), is a major genetic risk factor for cardiovascular disease. It is unknown whether an inverse relationship exists between Lp*(a)* and β-cell function (BCF), as for LDL-cholesterol (LDL-C) lowering by statins.

We therefore assessedthe cardiometabolic phenotype of 340 men with type 2 diabetes mellitus (T2DM) in relation to Lp*(a)*, focusing on BCF and hyperbolic product [BxS], which adjusts BCF to insulin sensitivity and secretion.

**Methods:**

Two groups were analyzed according to Lp*(a)* quartiles (Q): a (very-)low Lp(a) (Q1;*n* = 85) vs a normal-to-high Lp(a) group (Q2-Q4;*n* = 255).

**Results:**

In the overall cohort, mean Lp*(a)* was 52 nmol.L^−1^. Median Lp*(a)* was 6 nmol.L^−1^ (Q1) vs 38 nmol.L^−1^ (Q2-Q4). There were no differences between groups regarding age; education; diabetes duration; body mass index; body composition and smoking. Q1 had significantly worse glycemic control, higher systolic blood pressure, more severe metabolic syndrome, and more frequent hepatic steatosis. Insulin sensitivity was significantly lower (− 37%) in Q1, who also had lesser hyperbolic product (− 27%), and higher [BxS] loss rate (+ 15%). Q1 also had higher frequency (+31%) and severity (+20%) of atherogenic dyslipidemia. Microangiopathy and neuropathy were higher in Q1 (+ 34% and + 48%, respectively), whereas Q2-Q4 patients had increased macroangiopathy (+ 51%) and coronary artery disease (CAD; + 94%).

**Conclusions:**

Low Lp*(a)* appears both beneficial and unhealthy in T2DM. It is associated with unfavourable cardiometabolic phenotype, lesser BCF, poorer glycemic control, and increased microvascular damage despite being linked to markedly reduced CAD, suggesting that Lp*(a)-*related vascular risk) follows a J-shaped curve.

## Background

In recent years, a controversy in the general media has overstated the potential risks of statins, while minimizing the cardiovascular (CV) benefits of the class to reduce atherosclerosis. An unexpected positive aspect of this debate was a better understanding of the hyperglycemic effect of statins [1–3]. This impact, of little clinical significance, was often observed in large clinical trials, as new-onset diabetes and/or as modest depression of glycemic control in known diabetics. This hyperglycemic effect was variously ascribed to inhibition of islet *3-hydroxy-3-methylglutaryl-coenzyme A reductase*, lesser peripheral insulin sensitivity (IS), and/or decreased uptake of low-density lipoprotein cholesterol (LDL-C) by the β-cell secondary to reduction in circulating LDLs. Conversely, patients with familial hypercholesterolemia, whose baseline LDLs were exceedingly high for decades, are at lesser risk of developing type 2 diabetes mellitus (T2DM) [4].

Lipoprotein*(a)* (Lp*(a)*) is a variant LDL covalently attached to its specific apolipoprotein Apo(a), encoded by the LPA gene. The latter determines the number of pretzel-like domain-IV duplicates, inversely correlated with Lp*(a)* number, which, as a result, is genetically determined for each individual. Although Lp*(a)* is in itself a distinct subclass within LDLs, its physiological role(s) remain(s) unknown, despite the fact that elevated Lp*(a)* markedly increases atherothrombosis risk and incident CVD [5–11].

While it is established that statins and/or low LDL-C levels impair insulin secretion in non-diabetic, pre-diabetic and T2DM subjects, it is unclear whether the same relationship exists with respect to Lp*(a)*. As early as 1976, Dahlën & Berg found that Lp(a) modulates glucose and insulin levels, observations also reported later [5, 7]. Lp*(a)* was inversely associated with new-onset diabetes in the general population, as shown in the *Women Health* and the *Copenhagen City Heart* studies, independent of body mass index (BMI); glycated hemoglobin (HbA_1c_), or triglycerides (TG) [8], the inverse association being ascribed to lesser insulin resistance [6, 9].

More fundamentally, there has been no specific investigation of the relationship between Lp*(a)* and β-cell function (BCF), in particular it is unknown whether, as in the case of common LDLs, low numbers of Lp*(a)* particles are linked to β-cell function loss in T2DM. In this context, we analyzed the cardiometabolic phenotype of T2DM men in relation to Lp*(a)*, focusing in particular on BCF, hyperbolic product (which adjusts BCF to insulin sensitivity (IS)), and secular loss of insulin secretion.

## Methods

### Study design

The study was retrospective and included 340 adult Caucasian males with T2DM, followed by the same physician and coauthor of this study (MPH) at the Cliniques universitaires St-Luc (Brussels) between January 2010 and December 2016. Exclusion criteria included patients chronically treated with medications that could substantially change IS or BCF, including systemic or topical corticosteroids, antiretroviral drugs, immune-modulatory drugs, and anti-psychotics. Were also excluded patients with chronic inflammatory diseases, cancer or major organ failure (respiratory, heart, and liver). Two groups were analyzed in parallel according to quartiles (Q) of Lp*(a)*: a Q1 group (*n* = 85) vs a group combining Q2; Q3 and Q4 patients (*n* = 255).

### Patients characteristics

The following socio-demographic and clinical variables were recorded: age; highest educational attainment (as proxy for socio-economic status) based on four categories: (i) secondary school with leaving certificate (no graduation); (ii) school leaving certificate (with graduation); (iii) further education, but no degree; and (iv) university degree or similar, with highest educational attainment dichotomized as lower [(i) + (ii)] vs higher [(iii) + (iv)]; diabetes duration; presence of 1st-degree familial history (mother and/or father and/or siblings) for DM, and/or for familial history of EOCHD (early-onset coronary heart disease), defined as occurrence of a 1st CVD event < 55 years (men) and < 65 years (women), with the exclusion of familial hypercholesterolemia; smoking history; and habitual ethanol intake. Hypertension prevalence was defined as systolic blood pressure (BP) ≥ 140 mmHg and/or diastolic BP ≥ 90 mmHg and/or current treatment with BP-lowering drug(s) prescribed for treating high BP.

Weight, height, and body mass index (BMI) were determined, together with body fat and skeletal muscle mass (BodyFat Analyzer, Omron BF 500; Omron Healthcare Europe B.V., Hoofddorp, The Netherlands). Waist circumference and conicity index were determined as surrogates for central/upper body adiposity (conicity index: waist circumference (m)/0.109√[weight(kg)/height (m)]). The presence of a metabolic syndrome (MetS) was defined by a score ≥ 3/5 for the following items: (i) impaired fasting glucose or diabetes; (ii) hypertension; (iii) enlarged waist; (iv) hypertriglyceridemia; and (v) decreased high-density lipoprotein (HDL) cholesterol, according to the IDF-NHLBI-AHA-WHF-IAS-IASO harmonized definition [12]. Non-alcoholic fatty liver (NAFL) was based on the finding on abdominal ultrasonography by a trained radiologist of hepatorenal echo contrast and liver brightness, in the absence of etiological factors associated with liver steatosis, including excess ethanol intake].

Each subject underwent non-invasive combined assessment of BCF and -IS using the Homeostasis Model Assessment (HOMA-2, computer-based, version: http://www.dtu.ox.ac.uk), from triplicates means of fasting glucose and specific insulin levels obtained after an overnight fast and discontinuation of all glucose-lowering or glucose-sensitizing therapies for 24 h (48 h in case of insulin glargine and long-acting sulfonylureas and 2–28 days in case of GLP-1-RA with short/long duration of action). For patients treated with pioglitazone, HOMA was performed prior to introduction of this long-acting insulin-sensitizer [13, 14].

Values of insulin secretory capacity ([B]; normal value 100%) were plotted as a function of IS ([S]; normal value 100%), defining a hyperbolic product area [B × S] (unit: %^2^; normal: 100%, corresponding to 10^4^%^2^), representing the true, underlying BCF. [BxS] loss over a subject’s lifespan (ie. the secular loss of hyperbolic product ([BxS] loss rate; %.year^−1^) was obtained by dividing the absolute loss of [BxS] already incurred, the value of which corresponds to 100% - current [BxS] value (%), by a subjects’ age (year) at the time of HOMA-modeling. This simple formula (100%-[BxS])/age) provides an estimate of annual [BxS] loss rate, based on the assumptions that (i) any newborn subject, including those who develop T2DM later, starts its life with a [BxS] value of 100% conferring normal carbohydrate homeostasis, and (ii) that the loss of [BCF] is, for the most part, linear over the years [15, 16].

### Ongoing therapies and comorbidities

Current medications were recorded: glucose-lowering drugs (metformin; sulfonylureas/glinides; pioglitazone; dipeptidyl peptidase-4 inhibitors (DPP-4-I); glucagon-like peptide-1 receptor agonists (GLP-1-RA); insulin); and CV drugs [BP-lowering agents; aspirin (as antiplatelet agent); lipid-modifying drugs (LMD): statins; fibrates and/or ezetimibe]. Estimated glomerular filtration rate (eGFR) was calculated using the *Modification of Diet in Renal Disease* equation [17]. Normo, microalbuminuria and macroalbuminuria were defined as urinary albumin excretion < 30 (normo-), 30–299 (micro-) and ≥ 300 μg.mg creatinine^−1^.1.73 m^2^ (macro-), from first-morning urine sample. Diabetic retinopathy and nephropathy were defined using ICD-9-CM diagnoses and procedure codes. The presence of a peripheral neuropathy was based on clinical examination (knee and ankle reflexes; Semmes-Weinstein monofilament test) and/or electromyography. Eye visual examinations by an experienced ophthalmologist and/or fluorescein angiography were performed to diagnose retinopathy.

Coronary artery disease (CAD) diagnosis was based on medical history (myocardial infarction, angioplasty, stenting, revascularization surgery and/or significant coronary stenosis confirmed by angiography), systematic review of all procedures, and/or screening (exercise testing; echocardiography; magnetic resonance imaging; or other subclinical disease imaging techniques). Cerebrovascular disease was defined as a history of stroke (*UK Prospective Diabetes Study* criteria: any neurological deficit ≥ 1 month, without distinction between ischemic, embolic and haemorrhagic events) and/or transient ischaemic attack [18]. Peripheral artery disease (PAD) was defined by a medical history of lower-limb(s) claudication and/or clinical or imaging evidence for ischemic diabetic foot, angioplasty, stenting, revascularization surgery and/or lower-limb artery stenosis at Doppler ultrasonography and/or angiography.

### Laboratory values

HbA_1c_; fasting lipids (total cholesterol (C), HDL-C, triglycerides; LDL-C (computed using Friedewald’s formula), Lp*(a)*, and non-HDL-C (by subtracting HDL-C from total C)); apolipoproteins A-I (apoA-I) and B_100_ (apoB_100_); _hs_CRP; thyroid-stimulating hormone (TSH); serum total and free testosterone; sex hormone-binding globulin (SHBG); and albuminuria were determined by routine methods. Total cholesterol and TG were determined using SYNCHRON^®^ system (Beckman Coulter Inc., Brea, CA). HDL-C was determined with ULTRA-N-geneous^®^ reagent (Genzyme Corporation, Cambridge, MA). ApoA-I and apoB_100_ were determined with immunonephelometry on BNII Analyzer® (Siemens Healthcare Products GmbH, Marburg (Germany). Lp*(a)* concentration was determined by turbidimetric analysis of fresh plasma samples (Tina-quant^®^ Lipoprotein(a) Gen. 2 on Cobas c 502 module analyzer (Roche Diagnostics SA, Rotkreuz (Switzerland); measurement range: 7–240 nmol/L, regardless of isoforms; threshold value for Lp*(a)*-related CV risk increase > 75 nmol/L). Mean (SD) local reference values for Lp*(a)* obtained from a group of 50 healthy Caucasian male volunteers were 40 (37) nmol.L^−1^ (median 26 [IQR 10–72] nmol.L^−1^; range 7–124 nmol.L^−1^).

Atherogenic dyslipidemia (AD) was defined as the combination of low HDL-C (< 40 mg.dL^−1^) and high fasting TG (≥ 150 mg.dL^−1^) based on the MetS definition’s cutoffs for non-LDL lipids. AD prevalence, was established from the combined occurrence of low HDL-C plus high TG, from last available fasting TG and HDL-C measurements prior to LMD implementation [LMD(s)-treated patients], or from current fasting TG and HDL-C [LMD-naïve patients], respectively. AD severity was quantified as continuous variable using *log*(TG)/HDL-C ratio [19, 20].

Results are presented as means (± 1 standard deviation (SD)) or as median [interquartile range (IQR)]. The significance of differences between means was assessed by Student’s t test or by alternate Welch’s test for data sets with significant differences in SDs, and by Fisher’s Exact test for differences in proportions. Results were considered significant or non-significant (NS) for p < or ≥ 0.05 respectively.

## Results

### Patients characteristics

There were no differences between Q1 and Q2–4 patients regarding age; education (lower vs higher: 45% vs 55% (Q1) and 44% vs 56% (Q2–4)); diabetes duration; family history (DM and/or EOCHD); and smoking (Table 1). Habitual ethanol intake and self-reported leisure-time physical activity were also similar between groups (*not shown*). Glycemic control was on average above target, with mean HbA_1c_ in the overall population at 59 (11) mmol.mol^−1^. Q1 patients had significantly worse glycemic control, at 61 (13) vs. 58 (10) mmol.mol^−1^ in Q2–4 (p 0.0276). Patients were predominantly obese in the overall study population, with central adiposity. There were no differences between groups in terms of BMI, waist circumference, body composition (fat mass, visceral fat and skeletal muscle mass), and adiposity distribution (conicity index). Systolic BP was higher (by an average 5 mmHg) in Q1 patients, in whom the prevalence of hypertension was increased by 10%, and close to 100%. The prevalence of MetS was high and similar in both groups; however, Q1 patients had higher MetS severity score (8% relative increase). They also had a markedly increased frequency of non-alcoholic hepatic steatosis (33% relative increase).

**Table 1:** Patients characteristics

		1st Quartile Lp(a)	Quartiles 2–4 Lp(a)	*P*
*n*		*85*	*255*	~
age	years	68 (10)	67 (12)	*NS*
diabetes duration	years	18 (9)	16 (9)	*NS*
HbA_1c_	mmol.mol^−1^	61 (13)	58 (10)	*0.0276*
diabetes family history	%	48	50	*NS*
EOCHD family history	%	12	10	*NS*
smoking^a^		35 - 46 - 19	33 - 49 - 18	*NS*
hypertension	%	98	89	*0.0140*
systolic blood pressure	mm Hg	144 (21)	139 (19)	*0.0416*
body mass index	kg.m^−2^	29.0 (5.0)	28.8 (5.3)	*NS*
waist circumference	cm	105 (13)	105 (14)	*NS*
fat mass	%	26.7 (6.0)	25.9 (6.1)	*NS*
visceral fat	0–30 score	14 (4)	14 (5)	*NS*
skeletal muscle mass	%	32.9 (2.8)	33.2 (3.1)	*NS*
conicity index	m^2^.kg^−1^	1.36 (0.07)	1.36 (0.08)	*NS*
metabolic syndrome	%	86	80	*NS*
	0/5 to 5/5	3.9 (1.1)	3.6 (1.1)	*0.0301*
hepatic steatosis	%	92	69	*<0.0001*

Results are expressed as means (1 SD) or proportions (%). ^a^never-former-current; *EOCHD* early-onset coronary heart disease, *HbA1c* glycated haemoglobin, *Lp(α)* lipoprotein(α), *NS* not significant

### Cardiometabolic phenotype

For all patients, mean insulin sensitivity was lowered (56% of normal), indicative of substantial insulin resistance (Table 2). Fasting insulinaemia was higher (by 36%) in Q1 patients, whose insulin sensitivity was significantly lower (by a relative 37%) compared to Q2–4 patients (*p* < 0.0001). In the general cohort, the hyperbolic product ([BxS]; a measure of residual β-cell function) was markedly reduced (28.1%), the mean annual loss of [BxS] being 1.32%.

**Table 2:** Cardiometabolic phenotype & therapies

		1st Quartile Lp(a)	Quartiles 2–4 Lp(a)	*P*
*n*		*85*	*255*	~
insulinaemia	pmol.L^−1^	136 (93)	100 (70)	*0.0014*
insulin sensitivity	%	40 (26)	62 (43)	*< 0.0001*
hyperbolic product [B x S]	%	22 (15)	30 (20)	*< 0.0001*
[B x S] loss rate	%.yr.^−1^	1.46 (0.65)	1.27 (0.49)	*0.0149*
βCS - metformin - TZD	%	37 - 76 - 7	45 - 71 - 3	*NS*
DDP-4-I / GLP-1-RA	%	15	29	*0.0100*
insulin	%	56	43	*0.0442*
	IU.day^−1^.kg^−1^	0.94 (0.86)	0.70 (0.44)	*NS*
ACE-I - ARB	%	44–34	36–28	*NS*
CCB - BB - diuretic	%	33 - 34 - 46	29 - 42 - 34	*NS*
aspirin	%	55	54	*NS*
anti-dyslipidemic drug(s)	%	86	85	*NS*
statin - ezetimibe	%	76–13	81–13	*NS*
fenofibrate	%	36	19	*0.0045*

Results are expressed as means (1 SD) or proportions (%). *ACE-I* angiotensin-converting enzyme inhibitor *ARB* angiotensin II type −1 receptor (AT1) blocker, *βCS* beta-cell stimulant, *BB* beta-blockers, *CCB* calcium-channel blocker, *DPP-4-I* dipeptidyl peptidase type 4 inhibitor, *GLP-1-RA* glucagon-like peptide 1 receptor agonist, *Lp(a)* lipoprotein(a), *TZD* thiazolidinedione, *NS* not significant

This hyperbolic product was further reduced in Q1 patients, whose residual β-cell function was decreased by 8% (absolute) and by 27% (relative) compared to Q2–4 patients. [BxS] loss rate was significantly more severe in Q1 patients (1.46%.year^−1^), ie a 15% faster loss rate (relative value).

With respect to glucose-lowering therapies, Q1 patients were significantly less often treated with incretin-based therapies, and more often (+ 30%) treated with insulin. There were no differences between groups regarding metformin, β-cell stimulant and/or glitazone use. CV medications were used in similar proportions in the 2 groups, except for a markedly increased use of fenofibrate (+ 89%) by Q1 patients (Table 2).

### Lipids and lipoproteins

Table 3 shows the lipids and lipoproteins values of the Q1 and Q2–4 groups. For the overall cohort, mean Lp*(a)* was 52 (73) nmol.L^−1^ (median 24 nmol.L^−1^; IQR 24–69 nmol.L^−1^; range 2–545 nmol.L^−1^). Median Lp*(a)* values were 6 (Q1); 11 (Q2); 38 (Q3); and 120 nmol.L^−1^ (Q4). More than 3/4 of the overall cohort had normal Lp*(a)*, ie < 75 nmol.L^−1^; a cutoff corresponding to the 78th percentile of the overall cohort. Median Lp*(a)* in Q2–4 patients was 38 nmol.L^−1^, which means that Q1 patients had median Lp*(a)* more than 6-fold lower than the median value for the 3 upper Qs.

**Table 3:** Lipids & lipoproteins

		1st Quartile Lp(a)	Quartiles 2–4 Lp(a)	*P*
*n*		*85*	*255*	~
lipoprotein(a) mean (SD)	nmol.L^−1^	7 (2)	68 (78)	~
lipoprotein(a) median [IQR]	nmol.L^−1^	6 [5–8]	38 [15–84]	~
cholesterol	mg.dL^−1^	150 (34)	155 (33)	*NS*
non-HDL-C	mg.dL^−1^	106 (36)	109 (33)	*NS*
LDL-C	mg.dL^−1^	70 (29)	79 (28)	*0.0114*
apoB_100_	mg.dL^−1^	82 (23)	84 (21)	*NS*
LDL-C. apoB_100_^−1^		0.87 (0.28)	0.95 (0.25)	*0.0207*
triglycerides	mg.dL^−1^	199 (194)	154 (105)	*0.0394*
HDL-C	mg.dL^−1^	44 (15)	46 (14)	*NS*
apoA-I	mg.dL^−1^	139 (30)	143 (23)	*NS*
atherogenic dyslipidemia	%	59	45	*0.0332*
log(TG).HDL-C^−1^		0.06 (0.03)	0.05 (0.02)	*0.0324*

Results are expressed as means (1 SD), medians [interquartile range (IQR)], or proportions (%). *apo* apolipoprotein, *C* cholesterol, *HDL* high-density lipoprotein, *LDL* low-density lipoprotein, *Lp(a)* lipoprotein(a), *TG* triglycerides, *NS* not significant

LDL-C level was significantly higher in Q2–4 (+ 13%), whereas total C; non-HDL-C; HDL-C; apoB_100_ and apoA-I levels were similar in the 2 groups. LDL size, estimated by the LDL-C/apoB_100_ ratio, was significantly reduced (− 8%) in Q1 patients, in whose both the frequency (+ 31%) and severity (+20% *of log*(TG)/HDL-C) of AD were significantly increased (Table 3). Regarding non-lipid laboratory values, there were no differences between groups with respect to _hs_CRP; SHBG; total and free testosterone; and TSH (*not shown*). In a multiple regression analysis taking into account potentially confounders of Lp*(a)* and glycemic/metabolic control (age; duration of diabetes, glomerular filtration rate; BMI; sedentarity; waist; insulin resistance; atherogenic dyslipidemia; HbA_1c_), no relationship was found but for a very modest effect of age (*r* ^2^ = 0.0121; *p* 0.039).

### Comorbidities

Damage to target organs is described in Table 4. Overall microangiopathy was markedly and significantly more prevalent in Q1 patients (+ 34%), with microangiopathy frequencies within Q2 to Q4: 39% (Q2); 49% (Q3), and 52% (Q4; NS for trend). Retinopathy frequency was increased by 20% in Q1 relative to Q2–4, although the difference did not reach significance. Of all the quartiles, Q2 patients had the lowest prevalence in microangiopathies, and were on par with Q1 with respect to lower rates of overall macroangiopathy. Q1 patients had a significantly increased prevalence of neuropathy (+ 48%) vs the frequencies within Q2 to Q4: 18% (Q2); 28% (Q3); and 31% (Q4; p NS for trend). There was no difference between groups with regard to eGFR and (micro)albuminuria prevalence or severity. On the other hand, for large vessels, Q2–4 patients showed a markedly increased prevalence in overall macroangiopathy (+ 51% relative; + 15% absolute), more specifically CAD, which was increased by + 94% (relative) and + 17% (absolute). Overall macroangiopahy frequencies within Q2 to Q4 were: 29% (Q2); 52% (Q3), and 52% (Q4; p for trend 0.0034), with CAD prevalence of 20% (Q2); 40% (Q3); and 42% (Q4; p for trend 0.0021).

**Table 4:** Cardiovascular complications

		1st Quartile Lp(*a*)	Quartiles 2–4 Lp(*a*)	*P*
*n*		*85*	*255*	~
microangiopathy	%	62.4	46.7	*0.0127*
retinopathy	%	27.1	22.5	*NS*
peripheral polyneuropathy	%	37.6	25.4	*0.0376*
eGFR	mL.min^−1^.1.73 m^2^	76 (25)	75 (27)	*NS*
albuminuria	mg.g creatinine-^1^	200 (497)	107 (357)	*NS*
normo - micro - macroalbuminuria	%	56 - 30 - 14	63 - 28 - 9	*NS*
macroangiopathy	%	29.4	44.3	*0.0158*
coronary artery disease	%	17.6	34.1	*0.0040*
cerebrovascular disease	%	8.2	9.0	*NS*
peripheral artery disease	%	8.2	11.8	*NS*

Results are expressed as means (1 SD) or proportions (%). *eGFR* estimated glomerular filtration rate, *Lp(a)* lipoprotein(a), *NS* not significant

## Discussion

The work aimed to determine whether T2DM patients with low Lp*(a)* have a variant phenotype, beyond the expectation of lesser risk of macrovascular damage. To do this, we compared patients with low to very-low Lp*(a)* (Q1) to a group of patients with normal (Q2 and Q3) or elevated (Q4) Lp*(a).* Our results show that low Lp*(a)* is associated with a specific cardiometabolic phenotype, combining higher systolic BP and hypertension; poorer glycemic control; greater microangiopathy and neuropathy frequency; and lesser macrovascular disease. They also had a more severe MetS score, and more frequent liver steatosis, the latter associated with hyperinsulinemia; AD prevalence, and smaller-denser LDLs.

Concerning carbohydrate homeostasis, Q1 patients were characterized by greater insulin resistance, poorer insulin secretion, and more pronounced BCF loss. Thenceforth, it is hardly surprising that more of them could not stay on oral glucose-lowering drugs only and were switched to insulin. These differences in glucose homeostasis are likely determinants of poorer glycemic control in (very) low Lp*(a)* patients, as diabetes duration was similar between groups.

As regards the increased prevalence of microangiopathy in (very) low Lp*(a)* patients, it is unlikely that it stemmed directly from the low level of the lipoprotein, based on the current paradigm of diabetic microvascular disease [21]. On the other hand, at least four aspects of the unfavorable cardiometabolic phenotype associated with low Lp*(a)* may have contributed to the genesis (or aggravation) of small vessel damage, namely (*i*) poorer glycemic control; (*ii*) higher BP; (*iii*) greater MetS score; and (*iv*) more prevalent and severe AD [19, 20, 22–25].

As expected, overall macroangiopathy, including CAD, was significantly more frequent in patients with higher Lp*(a)*, consistent with previous transversal or longitudinal studies in diabetic [10, 11] and non-diabetic patients [26–28]. Macroangiopathy prevalence increased already in the 3^rd^ quartile, suggesting enhanced vulnerability of large vessels at modestly high Lp*(a)* levels in diabetes. All this implies relativizing the current “normality” threshold for Lp*(a)* in diabetics. Regarding microvascular risk, it is rather linked to having a low (rather than a high) Lp*(a)*, with “normality” thereby considered beyond Q1. In contrast, the risk to large vessels appears linear over quartiles, with a marked increase in complications from Q3 onwards. As the median Lp*(a)* value of Q3 patients was well below the pathological threshold for non-diabetics (≥75 nmol.L^−1^), it seems obvious that the macrovascular cutoff should be revised downwards in T2DM. If one takes the vascular system as a whole (all vessel sizes combined), it becomes clear that the risk linked to Lp*(a)* in diabetes follows a J curve that is conditional on the size of the vessels involved, with a rise in small vessels risk at (very) low levels, and, as expected, higher macrovascular risk (especially CAD) at increasing Lp*(a)* levels.

The study population was exclusively male and Caucasian, which restricts the applicability of our findings in terms of gender and ethnicity. Another limitation of this study is related to the transverse design, which does not formally establish the direction of causality of the reported associations. The lack of association between Lp*(a)* levels and a series of cardiometabolic variables in multiple regression analysis is consistent with literature data showing that few non-genetic/acquired determinants are able to influence Lp(a) level in a given individual, with the exception of severe renal impairment or certain drugs, such as niacin or proprotein convertase subtilisin/kexin type 9 inhibitors. Thus, the stability of Lp*(a)* level over time is such that the observed associations are likely to be linked to Lp*(a)*, and not that Lp*(a)* levels would have been modulated by an unfavorable phenotype or the presence of vascular complications.

## Conclusions

Having a low level of Lp*(a)* appears both beneficial and unhealthy in T2DM. At the microvascular level, a low rate is associated with lesser ß-cell function, poorer glycemic control, and increased microvascular damage and neuropathy. On the other hand, the same low level of Lp*(a)* is associated, as in the general population, with a reduced prevalence of macrovascular disease, despite a less favorable cardiometabolic phenotype. This suggests that the overall vascular risk associated with Lp*(a)* follows a J-shaped curve in the particular T2DM population when the vascular system is being studied as a global target organ, all sizes of vessels combined.

## Abbreviations

AD: Atherogenic dyslipidemia
ApoA-I: Apolipoprotein A-I
apoB: apolipoprotein B_100_
[B]: HOMA-measured BCF
BCF: β-cell function
BMI: Body mass index
BP: Blood pressure
[BxS]: Hyperbolic product between β-cell function and IS
C: Cholesterol
CAD: Coronary artery disease
CHD: Coronary heart disease
CRP: C-reactive protein
CV: Cardiovascular
CVD: Cardiovascular disease
DM: Diabetes mellitus
DPP4-I: Dipeptidyl peptidase-4 inhibitor
eGFR: Estimated glomerular filtration rate
EOCHD: Early-onset CHD
GLP-1-RA: Glucagon-like peptide-1 receptor agonist
HbA_1c_: Glycated hemoglobin
HDL: High-density lipoprotein
HDL-C: High-density lipoprotein cholesterol
HOMA: Homeostasis model assessment
IS: Insulin sensitivity
LDL: Low-density lipoprotein
LDL-C: Low-density lipoprotein cholesterol
LMD: Lipid-modifying drug
Lp(a): Lipoprotein(a)
MetS: Metabolic syndrome
NAFL: Non-alcoholic fatty liver
non-HDL-C: non-high-density lipoprotein cholesterol
NS: Non-significant
PAD: Peripheral artery disease
Q: Quartile
[S]: HOMA-measured insulin sensitivity
SD: Standard deviation
SHBG: Sex hormone-binding globulin
T2DM: Type 2 diabetes mellitus
TG: Triglycerides (triacylglycerols)
TSH: Thyroid- stimulating hormone
